# Community-directed vector control to supplement mass drug distribution for onchocerciasis elimination in the Madi mid-North focus of Northern Uganda

**DOI:** 10.1371/journal.pntd.0006702

**Published:** 2018-08-27

**Authors:** Benjamin G. Jacob, Denis Loum, Thomson L. Lakwo, Charles R. Katholi, Peace Habomugisha, Edson Byamukama, Edridah Tukahebwa, Eddie W. Cupp, Thomas R. Unnasch

**Affiliations:** 1 Department of Global Health, College of Public Health, University of South Florida, Tampa, FL United States of America; 2 Nwoya District Local Government, Nwoya, Uganda; 3 Vector Control Division, Ministry of Health, Kampala, Uganda; 4 Department of Biostatistics, School of Public Health, University of Alabama at Birmingham, Birmingham, AL United States of America; 5 The Carter Center, Uganda office, Kampala, Uganda; 6 Department of Entomology and Plant Pathology, Auburn University, Auburn, AL, United States of America; University of Buea, CAMEROON

## Abstract

**Background:**

Onchocerciasis a neglected tropical disease that historically has been a major cause of morbidity and an obstacle to economic development in the developing world. It is caused by infection with *Onchocerca volvulus*, which is transmitted by black flies of the genus *Simulium*. The discovery of the potent effect of Mectizan (ivermectin) on *O*. *volvulus* microfilariae and the decision by its manufacturer to donate the drug for onchocerciasis spurred the implementation of international programs to control and, more recently, eliminate this scourge. These programs rely primarily on mass distribution of ivermectin (MDA) to the afflicted populations. However, MDA alone will not be sufficient to eliminate onchocerciasis where transmission is intense and where ivermectin MDA is precluded by co-endemicity with *Loa loa*. Vector control will likely be required as a supplemental intervention in these situations.

**Methodology/Principal findings:**

Because biting by the black fly vectors is often a major nuisance in onchocerciasis afflicted communities, we hypothesized that community members might be mobilized to clear the breeding sites of the vegetation that represents the primary black fly larvae attachment point. We evaluated the effect of such a community based "slash and clear" intervention in multiple communities in Northern Uganda. Slash and Clear resulted in 89–99% declines in vector biting rates. The effect lasted up to 120 days post intervention.

**Conclusions/Significance:**

Slash and clear might represent an effective, inexpensive, community- based tool to supplement ivermectin distribution as a contributory method to eliminate onchocerciasis and prevent recrudescence.

## Author summary

River blindness is one of the most important causes of morbidity in the developing world. The discovery of ivermectin and the decision by its manufacturer to donate the drug for river blindness spawned the development of programs to eliminate river blindness through mass treatment of afflicted populations. But ivermectin alone will not eliminate river blindness in much of Africa; additional interventions are necessary. We show that a simple community-based approach to controlling the black fly vector results in dramatic reductions in the vector population. Such a community-based approach to vector control will be compatible with the community-driven mass drug administration programs distributing ivermectin for onchocerciasis elimination in Africa. This should help reduce the time needed to obtain elimination and help prevent recrudescence once elimination is attained.

## Introduction

Onchocerciasis (river blindness) is a neglected tropical disease that is historically one of the most important causes of blindness worldwide [1]. The disease is caused by the human filarial parasite, *Onchocerca volvulus*. The major pathogenic manifestation of the infection, ocular damage leading to blindness, commonly affects individuals beginning in the second decade of life, disabling them as they enter their most productive period. Other manifestations include pruritis and severe skin disease. In hyper-endemic settings, the care of blinded individuals places a severe stress on the community, often leading to their dissolution [2]. The parasite is transmitted by black flies (primarily *Simulium damnosum sensu lato* in Africa) that develop as larvae in fast running rivers. Thus, transmission of the parasite is most intense in large river basins, rendering many such areas uninhabitable [2]. Unfortunately, these areas contain much of the fertile land in the African savanna. By preventing their use for agriculture, onchocerciasis has retarded economic growth in many of the poorest countries of Africa.

In the 1980s, Mectizan (ivermectin) was shown to be a potent microfilaricide against *O*. *volvulus* [3] and that mass treatment of an afflicted population could reduce parasite transmission [4, 5]. Merck, the manufacturer of ivermectin, announced that it would provide the drug free of charge for the treatment of onchocerciasis, “as much as needed for as long as needed” [6]. Because of this, several programs were begun to control or eliminate onchocerciasis, employing a strategy of ivermectin mass drug administration (MDA) to the afflicted communities. These included the African Programme for Onchocerciasis Control (APOC) in Africa and the Onchocerciasis Elimination Program of the Americas (OEPA). OEPA, employing a strategy of semi-annual distribution of ivermectin MDA has succeeded in eliminating onchocerciasis in four of the six formerly endemic countries in Latin America and has interrupted transmission in all but one binational focus in the region [7]. In Africa, studies conducted in Mali and Senegal [8] and Nigeria [9] demonstrated that APOC- administered annual ivermectin MDA eliminated onchocerciasis from some foci.

Despite these successes, ivermectin MDA is not a panacea in the struggle to eliminate onchocerciasis. Where vector densities are high, models suggest that ivermectin MDA alone will not be sufficient to interrupt transmission [10, 11]. These predictions have recently been supported by field data from Cameroon and Uganda, where transmission of *O*. *volvulus* continues despite 15 and 18 years of annual ivermectin MDA, respectively [12, 13]. Second, large portions of Central Africa afflicted with onchocerciasis are co-endemic for *Loa loa*. Individuals with high *L*. *loa* parasitemias are susceptible to developing severe side effects when given ivermectin [14]. This has complicated the use of ivermectin MDA in areas where *O*. *volvulus* and *L*. *loa* are co-endemic and has prevented the implementation of ivermectin MDA altogether in some areas.

Vector control as a tactic to combat onchocerciasis in Africa has a long history. The use of larvicides to eliminate adult black flies and block transmission of *Onchocerca volvulus* was first implemented in Kenya in1946 [15]. Elimination of the vector (*Simulium neavei*) was successful and follow-up studies conducted in 1964 confirmed that the parasite had been eliminated from that country [16]. This successful program was used as a model for the first international onchocerciasis control program in Africa, the Onchocerciasis Control Programme in West Africa, or OCP. The OCP was a large-scale, vertically integrated control program whose aim was to eliminate blinding onchocerciasis as a public health problem throughout eleven countries in West Africa through vector control. A great deal of public health value was accomplished by this landmark effort. Skin disease was significantly reduced, more than 200,000 cases of blindness were prevented and the size of the *O*. *volvulus* population was substantially decreased [17]. More recently, Uganda has demonstrated the power of utilizing a combination of vector control and ivermectin MDA. Uganda has used a strategy that combines vector control (local larviciding of breeding sites) with semi-annual MDA. This has resulted in the apparent interruption of transmission in 15 of the 17 foci in Uganda, a finding that has been confirmed in the Wadelai [18], Itwara [19] and Mt. Elgon [20] foci. These outcomes are similar to those reported from the island of Bioko where a combination of vector control followed by ivermectin treatment has resulted in elimination of the parasite [21].

These data suggest that vector control, used in combination with ivermectin MDA, is a powerful, synergistic strategy to eliminate onchocerciasis. However, traditional vector control of *S*. *damnosum s*.*l*. with larvicides has several drawbacks. Larvicides are expensive to apply, have potential detrimental environmental consequences and require technically trained individuals to calculate proper dosage. We hypothesized that community members might be mobilized to remove the trailing vegetation at the breeding sites that represent a primary attachment point for the black fly larvae as an alternative method to larviciding. Here we report a study whose overall objective was to determine if simple community-based removal of larval attachment sites could reduce the biting rate of the black fly vectors. We report the results of a number of different trials to evaluate the effect that such a "slash and clear" approach had upon the biting rates of the vector in onchocerciasis endemic communities in Northern Uganda. The data indicate that such a community-driven vector control initiative may be an effective and inexpensive tool to supplement ivermectin MDA and accelerate the effort to eliminate onchocerciasis from Africa.

## Materials and methods

### Selection of study sites

**Fig 1:**
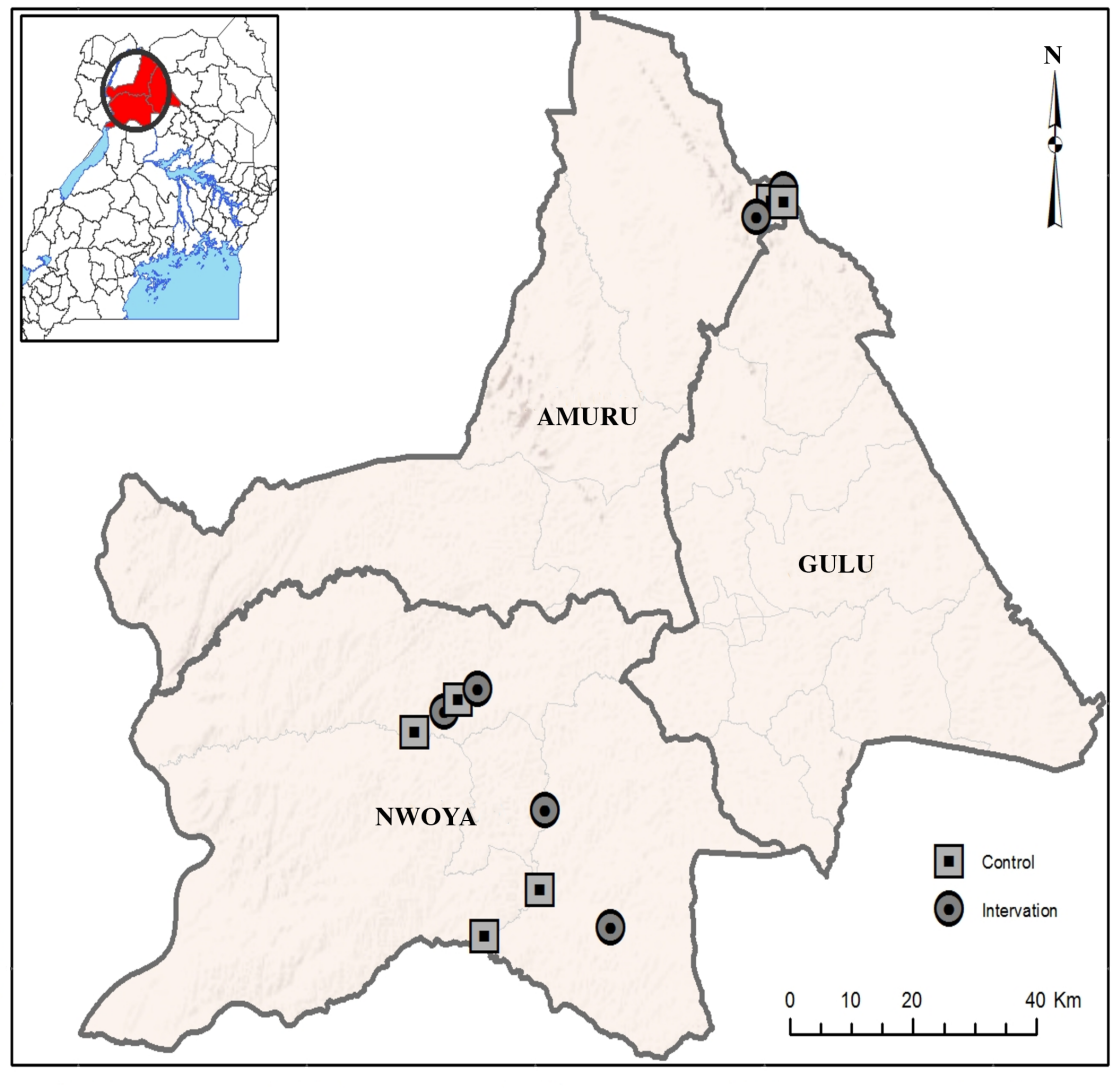
Location of study villages: As a first step in identifying study villages, potential *S*. *damnosum* breeding sites were identified by remote sensing using an extension of the black rock-rapid (BRR) signature model [24]. This extended model used a combination of the signatures characteristic of a rock substrate and a large gradient included in the BRR model [24] with specific spectral signatures for trailing vegetation, which are characteristic of the habitats at hyper-productive *Similium damnosum* s.s. breeding sites. An interpolation technique in ArcGIS was used to spatialize the calculated LULC values for the signatures derived from the residual forecasts of the bio-optical model. Villages were selected for potential inclusion in the study if they were located within 1km of a predicted breeding site and were separated by at least 15km from any other village selected for potential inclusion.

This study was conducted in communities located in Northern Uganda within the Madi-mid North focus of onchocerciasis (Fig 1). All communities were located in the districts of Amuru and Nwoya in the Madi-Mid North focus of Uganda. The vector of *O*. *volvulus* in this region is the savannah dwelling species *Simulium damnosum sensu stricto* [23]. While Uganda committed to a program of onchocerciasis elimination in 2007 [22] and began a program of country wide twice per year ivermectin distribution in onchocerciasis foci at that time, regular treatments were delayed in the Madi mid-North focus due to political unrest. Initial evaluations conducted prior to the start of the regular treatment program suggested that the region was hypo-endemic for onchocerciasis. The prevalence of skin snip positive individuals in the Amuru district was 1.5%, while no skin snip positive individuals were encountered in a survey of four villages in the Nwoya district. Regular twice per year treatments began in this region in 2010 and have been maintained since then. Mean therapeutic coverages in both districts have exceeded 90% in all treatment rounds since 2010.

The communities were each visited by the field team to validate their choice for inclusion in the study. Team members informally questioned the residents of the village about their knowledge of biting black flies, helping to determine if the flies represented a significant nuisance. Predicted breeding sites located within 1km of the village were validated by ground prospection to confirm the presence of *S*. *damnosum* larvae. As described below, all chosen communities were found to have similar biting rates prior to the start of the interventions.

### Implementation of the slash and clear technique

Selected villages were divided into pairs, with one village of each pair randomly assigned to the control group and one to the intervention group. Baseline collections using standard human landing techniques were carried out to establish the biting rate at each community. Following the baseline collections, young men (16–22 years of age) were recruited to carry out slash and clear in the intervention villages. The recruits were brought to the breeding sites located 1km upstream and downstream of the village and were instructed in the process of cutting the trailing vegetation from the water and throwing it on the river banks to dry, thereby killing the adherent black fly larvae. In the initial trials, two slash and clear cycles of intervention were conducted, with the first cycle conducted on days 8 and 9 of the study (i.e. after the 7-day baseline period) and the second conducted on days 19 and 20 of the study, thereby eliminating nearby larval substrates. Landing collections of adult flies were carried out daily throughout the study period (31 days total). In the long-term studies, collections were carried out daily throughout the first 20 days of the study, and twice per week thereafter. All community members, including the individuals participating in the study, were given ivermectin twice per year as part of the Uganda Onchocerciasis Elimination Program of the Uganda Ministry of Health.

### Statistical analysis of collection data

The number of flies collected in the intervention and control communities were compared at day 8 (at the start of the intervention), at day 18 (at the start of the second intervention) and at the end of each trial. Fly counts in the control communities at the start and end of the first two trials were also compared. The data were analyzed using a basic linear model that treated the river as a blocking effect and treatment type as the variable of interest. The basic factorial design model had the form

count=rivertreatmentriver*treatment

Because the data were counts, a negative binomial distribution model was used with SAS PROC GENMOD. A complete description of the statistical analysis and the results may be found in the S1 Supplemental Material.

## Results

**Fig 2:**
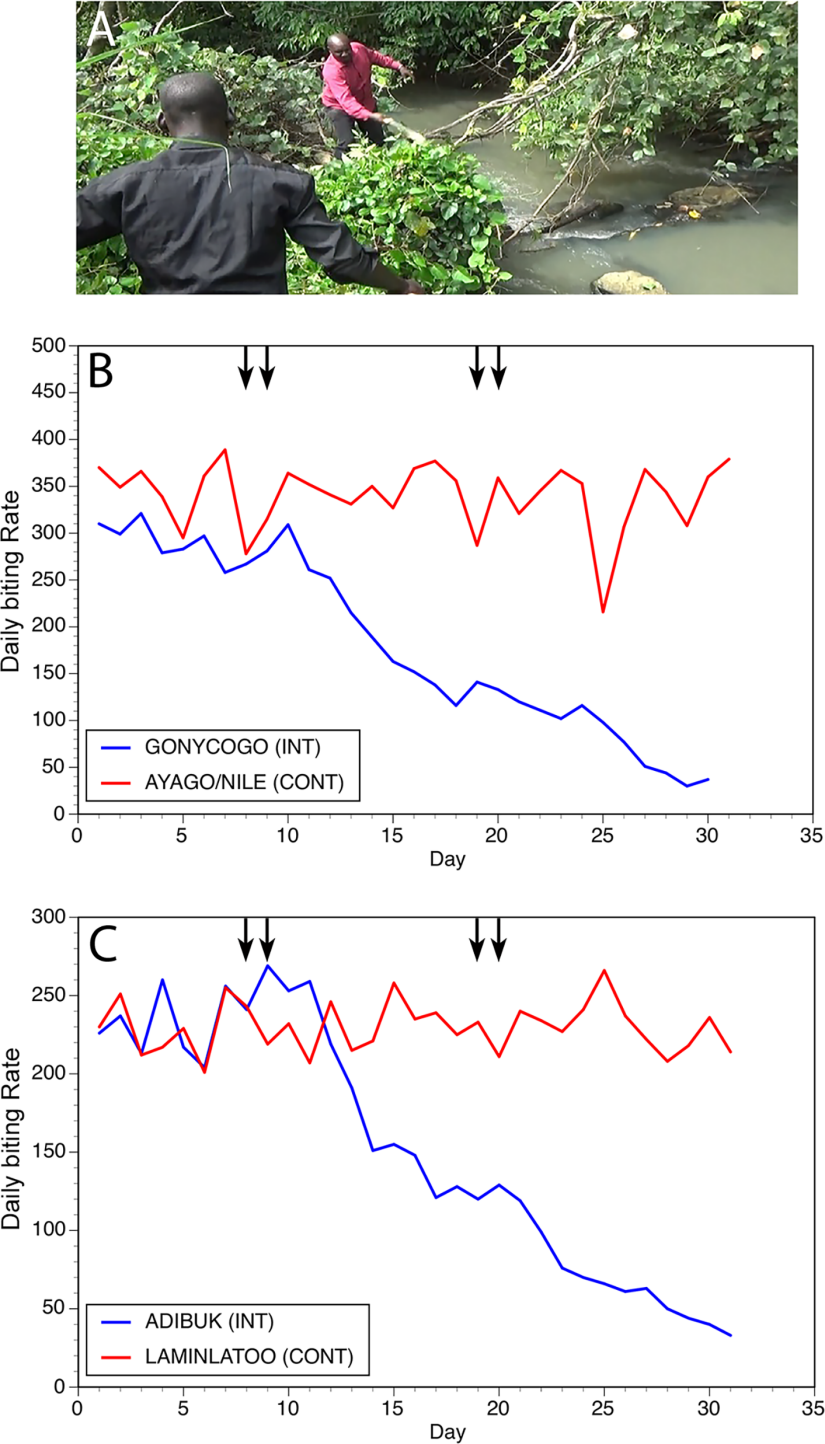
**Results of slash and clear interventions carried out on the Ayago river:** Panel A: Vegetation clearance at a typical breeding site on the Ayago. Panel B: Daily biting rates before and following interventions at the first matched pair of communities. Panel C: Daily biting rates before and following interventions at the second matched pair of communities. In Panels B and C, INT = intervention community and CONT = control community. Vertical arrows indicate days upon which slash and clear activities were carried out.

The initial trial of the slash and clear intervention was carried out on breeding sites near the villages of Gonycogo and Adibuk, located along the Ayago river, a small river in North Central Uganda (Fig 1). The villages of Laminlatoo and Ayago/Nile, also located along the Ayago, served as control communities. In all cases, the village inhabitants were found to be acutely aware of the extreme nuisance posed by blood-feeding black flies. The initial trials commenced on May 9, 2015, in the beginning of the rainy season in Northern Uganda. Trailing vegetation was removed from all breeding sites located within 1km of the intervention villages on days 8 and 9 and 19 and 20 (Fig 2, Panel A). No interventions were conducted in the control villages. Daily biting rates in the control and intervention communities were not significantly different from one another prior to commencement of the interventions (p> 0.1). Fly biting rates were seen to decline beginning at day 16, 6 days following completion of the first intervention, and were significantly lower than those in the control villages by day 18 (Fig 2, Panels B and C; p <0.0001). Fly numbers continued to decline through the end of the study, at which point the mean biting rate in the intervention villages was 11% of the mean biting rate in the control villages (mean daily biting rate of 32 in the intervention villages versus 296.5 in the control villages; Fig 2, Panels B and C; p < 0.0001). No significant change in the biting rate was seen in the control villages throughout the trial period (Fig 2; p = 0.9).

**Fig 3:**
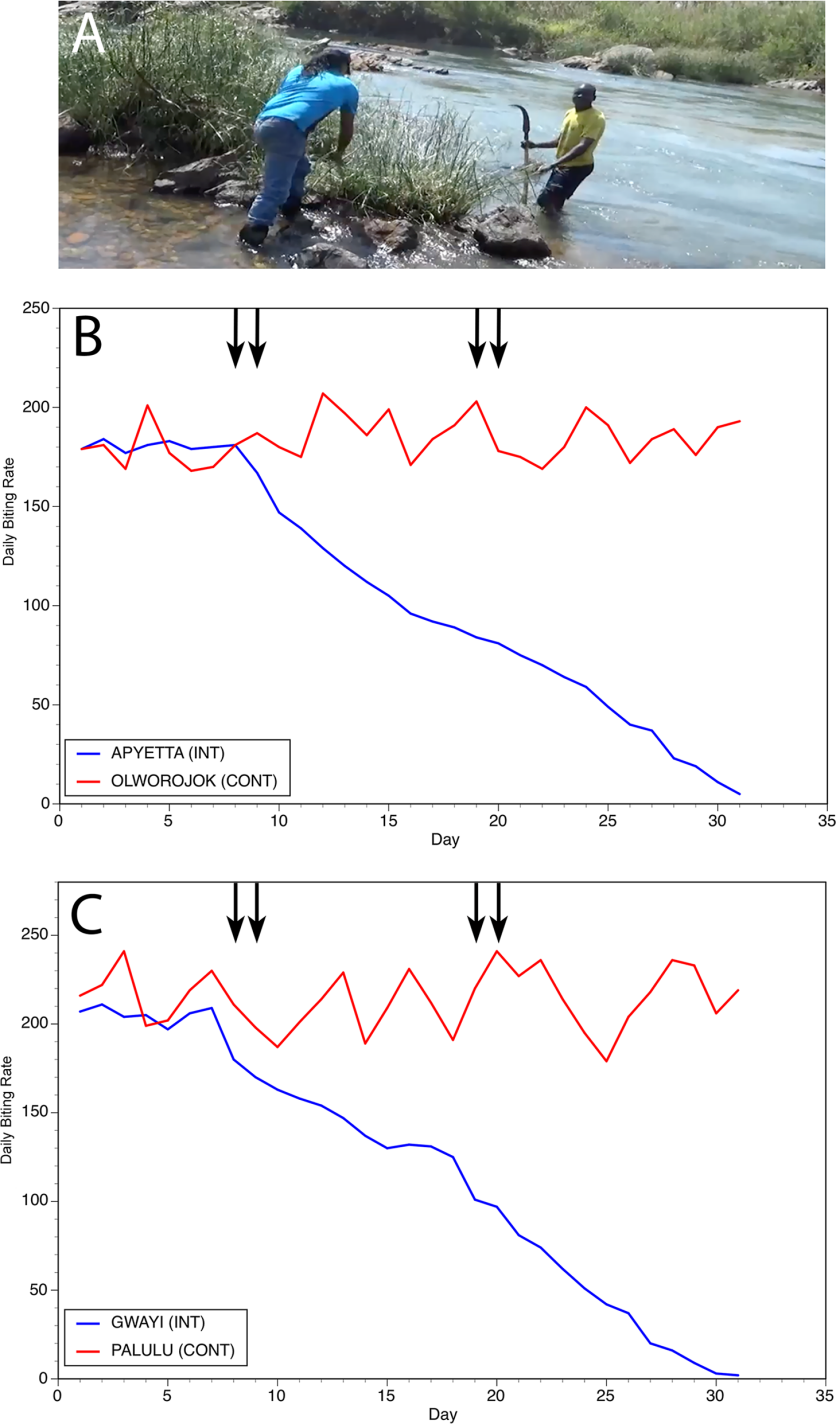
**Results of slash and clear interventions carried out on the Aswa river:** Panel A: Vegetation clearance at a typical breeding site on the Aswa. Panel B: Daily biting rates before and following interventions at the first matched pair of communities. Panel C: Daily biting rates before and following interventions at the second matched pair of communities. In Panels B and C, INT = intervention community and CONT = control community. Vertical arrows indicate days upon which slash and clear activities were carried out.

The Ayago river is a small stream, averaging 2m in width. Thus, it was of interest to determine if a similar approach could be used along larger rivers. A second study was carried out along the Aswa river, one of the largest rivers in the district, averaging 11m in width (Fig 3, Panel A). Again, four villages were identified using the methods described above. Two villages were assigned to the intervention group and two to the control group. Interventions were carried out on days 8 and 9 of the study and on days 19 and 20 (Fig 3, Panels B and C). This study was conducted in late August and early September 2015, which is during the peak fly biting season in this area. The results were similar to those obtained in the initial trial, with mean fly biting rates in the intervention communities declining to just 1% of the mean biting rates found in the control communities at the end of the study (mean daily biting rate in the intervention villages of 3.5 versus a mean daily biting rate in the control villages of 412; Fig 3, Panels B and C; p < 0.0001).

**Fig 4:**
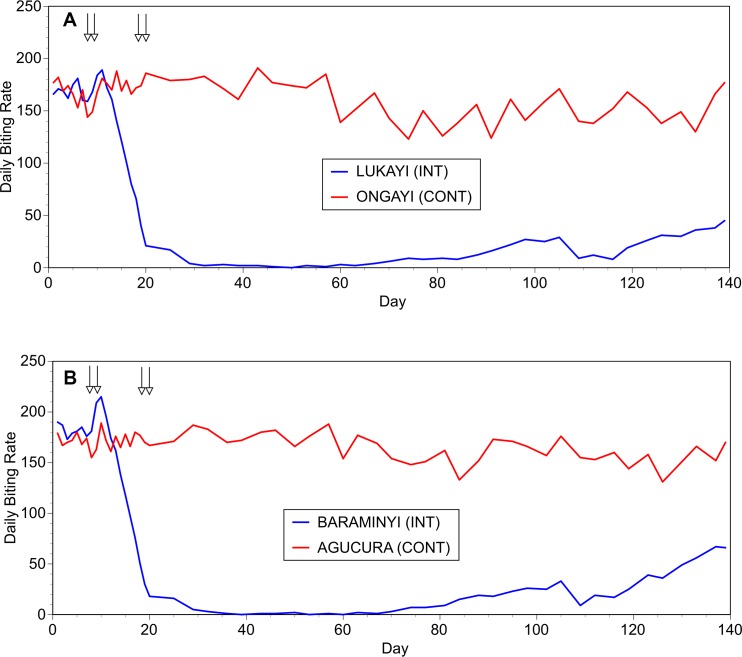
**Effect of the slash and clear interventions through a single rainy season:** Panel A: Daily biting rates before and following interventions at the first matched pair of communities. Panel B: Daily biting rates before and following interventions at the second matched pair of communities. In both panels, INT = intervention community and CONT = control community. Vertical arrows indicate days upon which slash and clear activities were carried out.

These initial studies were limited to approximately one month during the rainy season. Thus, it was of interest to determine how long the effect of the slash and clear intervention lasted. To begin to answer this question, a third trial was conducted on the Aswa river, which began on July 15, 2016, in the middle of the rainy season when fly numbers are high. Fly collections continued until November 30, 2016, near the end of the rainy season. Similar to the previous trials, the maximum reduction in the biting rate in the intervention villages was reached on day 29, where the mean biting rate in the intervention villages was 2.4% of that seen in the control villages (mean daily biting rate of 4.5 in the intervention villages versus a mean daily biting rate of 183.5 in the control villages; Fig 4, Panels A and B). This degree of reduction was maintained through day 67 of the study (September 19, 2016), at which point the fly numbers in the intervention villages began to slowly recover. At day 139 (the end of the trial), the mean biting rate in the intervention villages had reached 32% of the mean biting rate in the control villages (mean daily biting rate of 55.5 in the intervention villages versus 173.5 in the control villages; Fig 4).

**Fig 5:**
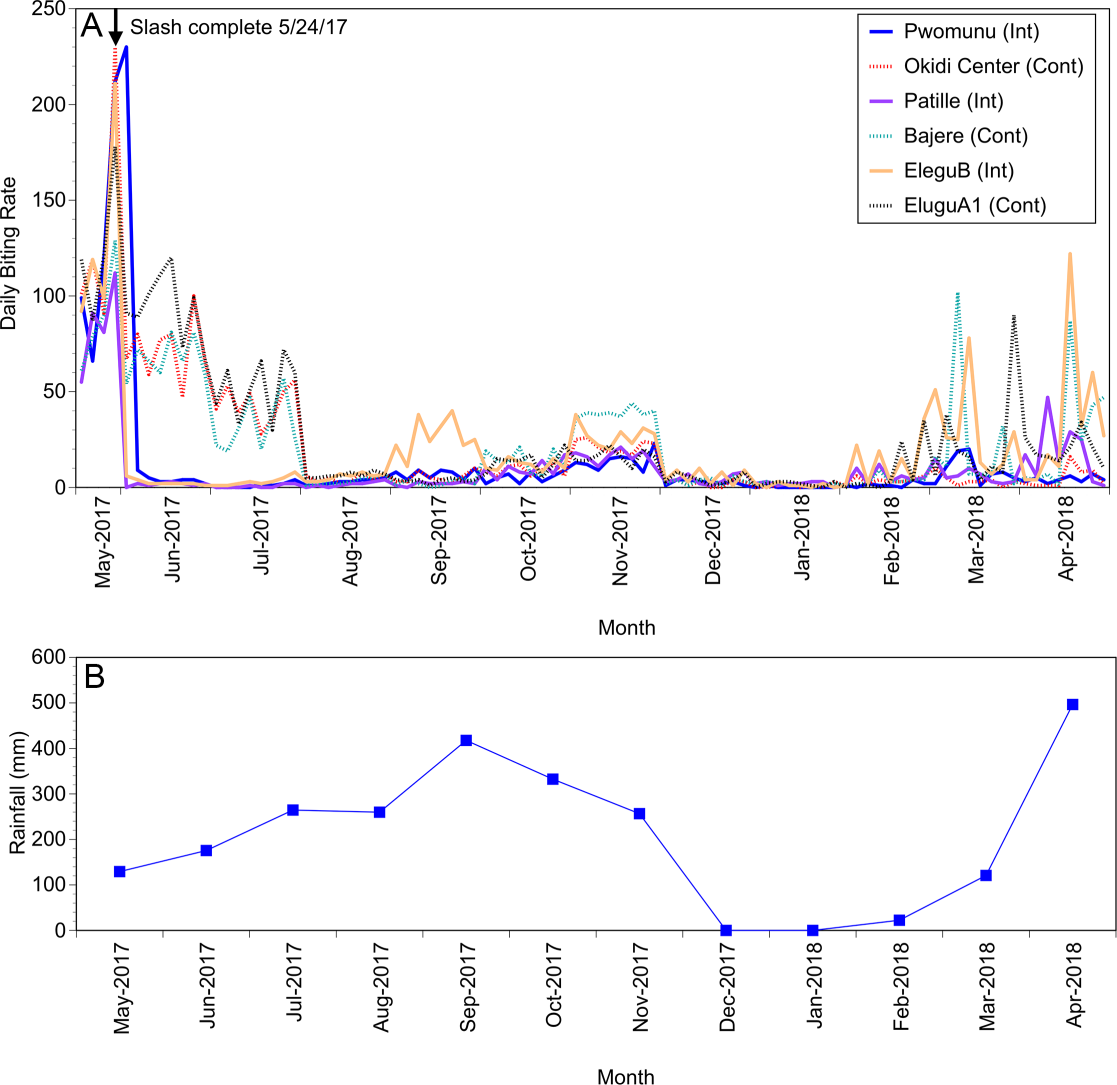
**Year-long analysis of the effect of a single slash and clear intervention:** Panel A: Daily biting rates before and following interventions at three control and three intervention communities. Solid lines represent intervention communities (Int) and dotted lines represent control (Cont) communities. Pre-intervention fly collections were carried out in May. Interventions were completed on May 24, 2017. Panel B: Monthly rainfall totals in the study area. Rainfall data were obtained from the Weather Atlas [25].

As the fly populations did not recover completely by the end of the first long-term trial, a second long-term trial was carried out beginning in May, 2017. The goal of this trial was two-fold; first to determine how long it took for the fly populations to recover to levels indistinguishable from the control sites, and second to obtain baseline data on the fly biting rates throughout the year. In this trial, a total of six communities were enrolled. Three were randomly assigned to the control group and three to the intervention group as before. A single slash and clear intervention was carried out towards the end of May, 2017 in the three intervention villages. The slash intervention was completed on May 24, 2017. Flies were collected twice per week from all villages for a total of one year (May, 2017-April, 2018). The daily biting rate in the intervention villages again fell dramatically, reaching 3.3% of the mean daily biting rate in the control villages on June 15, 2017 (a mean daily biting rate of 2.67 in the intervention villages versus a mean daily biting rate of 80.3 in the control villages; Fig 5, Panel A). This effect lasted through the end of July, when the mean daily biting rate in the intervention villages was 9.9% of that in the control villages (a mean daily biting rate of 4.67 in the intervention villages, versus a mean daily biting rate of 47.3 in the control villages; Fig 5, Panel A). In August, fly numbers fell precipitously in the control villages as a result of a flood that occurred in early August that removed much of the trailing vegetation from the control breeding sites. Fly numbers then began to recover in both the control and intervention sites in November, but then the fly numbers declined dramatically in all sites in December, marking the start of the dry season (Fig 5, Panel B). Fly numbers began to recover in all sites as the rains began to return in March, 2018 (Fig 5).

## Discussion

The data presented here suggest that removal of trailing vegetation by community members resulted in a dramatic reduction in daily vector biting rates in the Madi mid-North focus of onchocerciasis in Uganda. The maximum reductions we observed in the daily biting rate ranged from 89% to 99%, occurring roughly 20 days following the initial intervention. These results are similar to the only other study reporting an evaluation of a similar vegetation removal strategy conducted in Sudan in 1984 [26]. In that study, biting rate reductions of roughly 80% were reported. However, in that trial, vegetation removal was conducted in conjunction with larviciding, making it difficult to separate the effects of the two methods. This study demonstrates that vegetation removal alone resulted in a highly significant reduction in biting rates.

The effect of vegetation removal was quite long lasting. This is perhaps not surprising, as the vegetation clearance removed most or all of the trailing vegetation at the breeding sites, and we observed that the trimmed vegetation took months to recover. This is in contrast to larviciding, which, though it removes developing *S*. *damnosum* s.l. larvae in the breeding site does not alter the breeding site habitat. Thus, the breeding site can be rapidly re-populated by gravid adult flies that are not affected by larviciding [27]. In contrast, slash and clear removes vegetation substrates from the breeding habitat, limiting re-population until the substrates recover. Importantly, the duration of the effect of the slash and clear treatments means that if the goal is achieving a sustained reduction in biting rates, the removal process may need to be repeated only once every two months during the breeding season. Finally, slash and clear avoids the prospect of insecticide resistance, a fairly common phenomenon associated with long-term use of insecticides that significantly contribute to the cost and complexity of control programs [28]. As such, this approach fits well with a recent proposal that alternative vector control strategies should be considered to mitigate the impact of resistance [29].

We did not attempt to accurately assess the effect that the reduction in biting rate combined with MDA had on parasite transmission in these studies. This is because, as is the case for most previously endemic foci in Africa, the population in this area had received multiple treatments with ivermectin and transmission in the focus was dramatically reduced as a result. Furthermore, due to the success of the slash and clear process, insignificant numbers of flies were collected in the intervention sites once the interventions were performed. However, based on traditional studies where vector control drastically reduces annual biting rates and transmission, we believe that the impact would be significant on transmission of *O*. *volvulus* and would eventually threaten the existence of the parasite population.

We believe that there are three features inherent in the slash and clear method that may increase its potential for sustainability in the affected communities. First, in all the communities enrolled in the study, residents could readily identify the vector flies and all reported that biting from the flies represented a significant nuisance. The community members were motivated to become involved in any program that promised to reduce the number of biting flies plaguing them. Second, the expenses involved in carrying out the slash and clear interventions are minimal and involve materials already available and used routinely in the community (e.g. machetes and rubber boots). Thus, the investment required by the community to undertake and maintain a slash and clear intervention is minimal. Finally, we predict it is likely that as the fly densities return to a nuisance level, the community members will be motivated to independently conduct slash and clear interventions, thereby keeping biting rates low. Studies to test this hypothesis are currently underway.

It is notable that we observed dramatic reductions in the biting rate in all the intervention communities, despite the fact that only breeding sites located within a 1km radius of each village were targeted. This finding indicates that most black flies biting the community members were derived from nearby breeding sites. This suggests that targeting only the nearby breeding sites will be sufficient to dramatically reduce the biting rate in a given community, thereby likely reducing the annual transmission potential, a key epidemiological statistic. The data also suggest that as most of the flies take bloodmeals locally, the large majority of parasite transmission may also be driven by locally produced flies. This has implications for defining so called “transmission zones” when planning and implementing elimination programs. Previous studies have suggested that the savanna dwelling species of *Simulium damnosum* can travel long distances in West Africa, migrating on seasonal winds [30]. This suggests that transmission zones may be quite large, on the order of hundreds of kilometers in diameter. However, if most of the biting is the result of flies that are breeding in nearby breeding sites, it is likely that most of the transmission will be carried out by locally breeding flies, which would tend to shrink the effective size of the transmission zone and the contribution of migrating flies to overall transmission. Additional work will be necessary to quantify the contribution of migrating flies to the overall level of transmission.

Models of the dynamics of transmission of *O*. *volvulus* suggest that transmission intensity is strongly affected by the rate of host-vector contact [10, 11]. Thus, reducing vector densities can be an effective method of reducing or suppressing transmission [31]. In fact, the first large scale international program that eliminated blinding onchocerciasis as a public health problem, the Onchocerciasis Control Programme in West Africa (OCP), relied almost exclusively on vector control to reach their goal [32]. We hypothesize that the slash and clear approach to vector control may be applicable in at least three situations in the effort to eliminate onchocerciasis: 1. As a supplement to ivermectin MDA to reduce the time necessary to achieve elimination; 2. As an adjunct intervention to the selective use of ivermectin (employing the "test and not treat" strategy in areas co-endemic for *L*. *loa* and *O*. *volvulus*), and; 3. As a way to prevent recrudescence from occurring once onchocerciasis has been eliminated from a given focus. The effectiveness of slash and clear in these situations could be estimated by using the data generated in this study in models that are being developed to assist in the onchocerciasis control and elimination efforts in Africa [31]. Such studies are currently underway.

While the data presented here are very encouraging, it is likely that slash and clear will not be a panacea for every effort to eliminate onchocerciasis from Africa. First, these studies targeted the savanna dwelling species of *S*. *damnosum* s.l. and involved relatively small rivers. It is unlikely that this strategy will be as successful when applied in communities located near breeding sites in some of the large powerful rivers in Africa (such as the Nile). Furthermore, although the savanna dwelling species represent the major vectors of *O*. *volvulus* throughout most of sub-Saharan Africa, applying this method to foci in which some of the other sibling species of *S*. *damnosum s*.*l*. are vectors may prove to be more difficult. For example, breeding in forested onchocerciasis foci occurs in small streams, making the identification of all the breeding sites located near a community difficult. Additional studies will be necessary to evaluate the effect of slash and clear in such environments.

## Supporting information

S1 Supplemental Material (DOCX)
