# Supplementary material for: Community-directed vector control to supplement mass drug distribution for onchocerciasis elimination in the Madi mid-North focus of Northern Uganda
Source: PLoS Negl Trop Dis. 2018 Aug 27;12(8):e0006702. doi: 10.1371/journal.pntd.0006702 (PMC6128654; doi:10.1371/journal.pntd.0006702)
Supplement: S1 Supplemental Material — (DOCX) [file pntd.0006702.s001.docx]

**Supplemental Material**

Examination of the data presented in Figures 2 and 3 suggest that in the baseline collection periods the control and intervention villages exhibited very similar vector biting densities as assessed by the human landing collections. Thereafter, the number of flies caught in the intervention villages began to decline steadily in the intervention villages after the interventions were carried out, while the fly densities in the control villages remained fairly steady. This decline continued through the second intervention and through the end of the trials. Our objective is test the hypothesis that after treatment the fly densities in the intervention villages were significantly less than the densities in the control communities. The factors considered in the analysis of two trials were the rivers (Ayago and Aswa) on which the villages were located and the treatment condition (control or intervention). The Aswa and Ayogo trials were combined for the analysis and cross-sectional comparisons of the treatment counts were made at days 8, 18 and 31. The basic linear statistical model treated river as a blocking effect and treatment type as the variable of interest. The basic factorial design had the form

*count = river treatment river ∗ treatment*

Because the data were counts, a negative binomial distribution model was used with SAS PROC GENMOD.

As expected, there was no difference between the control and intervention collection counts on either river at day 8, the day interventions began to be carried out and the end of the baseline period (Table S1). However, there were significantly more flies caught on the communities located on the Ayogo river than in the communities on the Aswa river (p < 0.0001). In contrast, on day 18 (ten days after the interventions began) the number of flies collected in the intervention villages were significantly less than the number collected in the control villages

(Table S2). Once again, the number of flies collected in the control communities along the Ayogo River were significantly greater than the number collected in the control villages along the Aswa River ( p < 0.020). however, no difference was noted in the number of flies collected in the intervention villages along the two rivers (p = 0.844), reflecting the dramatic effect of the slash and clear treatments on overall biting fly densities in the intervention communities on both trials.

A similar pattern was seen at day 31, at the end of the trials. Once again, the number of flies collected in the intervention villages were significantly less than the number collected in the control villages in both trials (Table S3). However, at this time point, the number of flies collected in the control villages along the Ayogo River was not significantly different than the number of flies collected in the villages along the Aswa River (p = 0.19), while the number of flies collected in the intervention villages along the Ayogo were significantly greater than the number collected in the intervention villages along the Aswa (p < 0.001).

**Table S1: Comparisons of Least Square Means for Collections in Control and Intervention Communities on Day 8:**

| River | LS Mean Control | LS Mean Intervention | p-value for difference |
| --- | --- | --- | --- |
| Aswa | 196 | 180 | 0.259 |
| Ayogo | 260 | 254 | 0.685 |

**Table S2: Comparisons of Least Square Means for Collections in Control and Intervention Communities on Day 18:**

| River | LS Mean Control | LS Mean Intervention | p-value for difference |
| --- | --- | --- | --- |
| Aswa | 191 | 107 | 0.0010 |
| Ayogo | 290 | 122 | 0.0001 |

**Table S3: Comparisons of Least Square Means for Collections in Control and Intervention Communities on Day 31:**

| River | LS Mean Control | LS Mean Intervention | p-value for difference |
| --- | --- | --- | --- |
| Aswa | 206 | 4 | 0.0001 |
| Ayogo | 296 | 32 | 0.0001 |
